# Influence of cooking process on the content of water‐soluble B vitamins in rice marketed in Iran

**DOI:** 10.1002/fsn3.2690

**Published:** 2021-12-28

**Authors:** Mohammad Rezaei, Mahmood Alizadeh Sani, Mohsen Amini, Nabi Shariatifar, Mahsa Alikord, Majid Arabameri, Anita Chalipour, Reza Hazrati Reziabad

**Affiliations:** ^1^ Department of Environmental Health Engineering School of Public Health Tehran University of Medical Sciences Tehran Iran; ^2^ Department of Medicinal Chemistry Faculty of Pharmacy Drug Design and Development Research Center The Institute of Pharmaceutical Sciences (TIPS) Tehran University of Medical Sciences Tehran Iran; ^3^ Food Safety Research Center (salt) Semnan University of Medical Sciences Semnan Iran; ^4^ Food and Drug Administration Tehran University of Medical Sciences Tehran Iran

**Keywords:** cooking methods, food analysis, heatmap visualization, multivariate techniques, water‐soluble B vitamins

## Abstract

The aim of this study was to analyze the effect of cooking method on thiamin (B1), riboflavin (B2), and pyridoxine (B6) vitamin content of rice samples consumed in Iran by using high‐performance liquid chromatography technique. The amount of B1, B2, and B6 obtained ranged from 2.98 to 15.89, 1.15 to 22.19, and 0.96 to 4.44 μg/g, respectively, for the boiling method. In the traditional method, these vitamins had a concentration between 4.09 and 29.55, 4.87 and 16.19, and 1.52 and 12.18 μg/g, respectively. However, limit of detection (LOD) values for B1, B2, and B6 vitamins were 0.159, 0.090, and 0.041 μg/ml, respectively. Multivariate methods and heatmap visualization were applied to estimate the correlation among the type and amount of vitamins and cooking methods. According to heatmap findings, B1 and B6 vitamins and the cooking method had the closest accessions, representing that this variable had similar trends. Nevertheless, it can be concluded that the traditional cooking method can maintain more vitamins in rice samples.

## Graphical abstract

The amount of B1, B2, and B6 obtained ranged from 2.89 to 15.89, 1.15 to 22.19, and 0.96 to 4.44 mg/kg, respectively, for the traditional method. In the boiling method, these vitamins had a concentration between 4.09 and 29.55, 4.87 and 16.19, and 1.52 and 12.18 mg/kg, respectively. However, LOD values for B1, B2, and B6 vitamins were 0.159, 0.090, and 0.041, respectively. Multivariate methods and heatmap visualization were applied to estimate the correlation among the type and amount of vitamins and cooking methods.

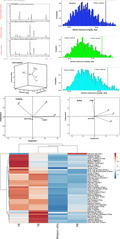

## 1 INTRODUCTION

Nowadays, from the perspective of food safety and security, and nutrition sciences, rice (*Oryza sativa* L.) is recognized as one of the most sustainable and important foods for the majority of the population and provides a considerable amount of energy required in the diet (Bresciani et al., 2021; Verma & Srivastav, 2020). Its annual consumption in most African and Asian nations such as Iran, India, Pakistan, and China has exceeded 36 kg (Shariatifar et al., 2020). Hence, due to the high consumption of this carbohydrate, it is considered a valuable source of vitamins and minerals for humans. Consequently, its inclusion in the diet can compensate for the nutritional deficiencies in consumers (Liu et al., 2019; Shariatifar, Rezaei, Sani, et al., 2020). Since different types of Iranian, Pakistani, and Indian rice are supplied and consumed all over the world, the type of variety, method of cultivation, changes in climatic conditions, water, and soil quality can affect the nutritional value of rice produced in terms of macro‐ and micronutrients (Shariatifar, Rezaei, Sani, et al., 2020).

In addition to the proposed environmental factors, processing conditions during the harvesting process (e.g., husking, polishing, and milling process), the type of cooking method, and the initial precooking processes also affect the quality and nutritional value of rice consumed (K.‐l. Liu et al., 2017; Liu et al., 2019; Zhu et al., 2019). Many studies have described that process effects such as washing and soaking rice as typical precooking processes or cooking with high water as well as cooking temperature play a considerable role in the micro‐ and macronutrient content of rice cooked (Shariatifar, Rezaei, Sani, et al., 2020; de Souza Batista et al., 2019; Zhu et al., 2019). Therefore, although washing and soaking rice before cooking will improve the quality of the final cooked rice, it can also eliminate and reduce dust on the rice grain surface, nutrients, starch, and water‐soluble vitamins (Liu et al., 2019). Previous research reported that a substantial amount of vitamins and mineral contents was wasted after washing, soaking, and cooking rice (Cheigh et al., 1977; Mihucz et al., 2010). It has also been shown that the rate of nutrient loss in rice soaked in warm water increases significantly (Jafar et al., 2008). Moreover, it has been indicated that soaking, washing, and cooking lead to a notable decrease in thiamine (B1) and riboflavin (B2) contents in cooked rice, and more washing and soaking time will cause further loss of these vitamins.

As regards rice, it is generally cooked in two ways: (i) traditional method (cooking rice with a constant amount of water without removing the water) and (ii) boiling method (cooking rice with extra water and then eliminating the water) (Shariatifar, Rezaei, Sani, et al., 2020); it is expected that the rate of loss of minerals and vitamins in the method of soaking with extra water will increase, as reported in previous studies (Liu et al., 2019; Shariatifar, Rezaei, Sani, et al., 2020). Therefore, considering that rice is a very important source of vitamins, especially thiamine (B1), riboflavin (B2), and pyridoxine (B6) as well as other minerals, and these vitamins play a vital role in physiological processes, macronutrient syntheses, metabolism, and prevent other disorders such as anemia, cancer, and coronary disease in humans (Descombes et al., 1993; Galán & Drago, 2014; Mahan & Raymond, 2016), as a result, measuring the remaining amount of these vitamins in cooked rice before consumption seems to be essential for a balanced diet.

Accordingly, the purpose of the study was first to measure the content of residual vitamins (vitamin B1 (thiamine), B2 (riboflavin), and B6 (pyridoxine)) in rice samples after cooking by both conventional and soaking methods by using high‐performance liquid chromatography with a UV detector and to investigate the effect of different enzymes (protease and amylase) on the release of vitamins from rice samples in the sample preparation phase. Second, multivariate test (one of the most prominent statistical techniques) is used to describe the interrelationships between variables and visualize data patterns, and heatmap is used to display similar or very different values to display the similar or vastly different expression status characteristic values.

## 2 MATERIALS AND METHODS

### 2.1 Chemicals

All reagents and standards were obtained from Merck (Darmstadt, Germany).

### 2.2 Sample collection

In this study, 22 samples from different Iranian, Pakistani, and Indian brands were purchased from markets in Iran.

### 2.3 Cooking rice methods

Rice samples were cooked by traditional and boiling cooking methods (Shariatifar, Rezaei, Sani, et al., 2020). For boiling cooking conditions, 200 g of each type of rice was added to boiled deionized water (1000 ml) in a Pyrex container. The samples were then boiled on a flame for 15 min. Afterward, the extra water was removed by a colander, and semi‐cooked rice samples were transferred to another container for the final cooking stage at 105°C for 1 h. In the traditional cooking method, 200 g rice was cooked with boiled water (400 ml) on a flame at 105°C for 1 h without extra water removal. The samples were immediately cooled, frozen at −18°C, until analysis.

### 2.4 Preparation of standard solutions

The number of B vitamins in the samples was quantified by comparison with the standard. Stock solutions of thiamin and riboflavin were made ready by dissolving 5 mg of the respective compound in 50 ml of deionized water (1 mg/ml), and a stock solution of pyridoxine was made ready by dissolving 5 mg of the respective compound in 100 ml (0.5 mg/ml).

### 2.5 Vitamin measurement

Portions of the rinsed and traditional rice samples were analyzed according to the chopped sample (5 g) method and were added to 30 ml of H_2_SO_4_ (0/1 M) and the contents were incubated (121°C) for 30 min. Then, they were adjusted to pH 7 with sodium hydroxide (0.1 N). Five milliliters of amylase enzyme (10%) and 5 ml of protease (10%) were added to each sample and incubated overnight at 35°C. The sample was diluted to 100 ml using deionized water and vortexed; then, 20 μl of the solution obtained through a micropore filter (0.45 μm) was analyzed using HPLC.

**FIGURE 1:**
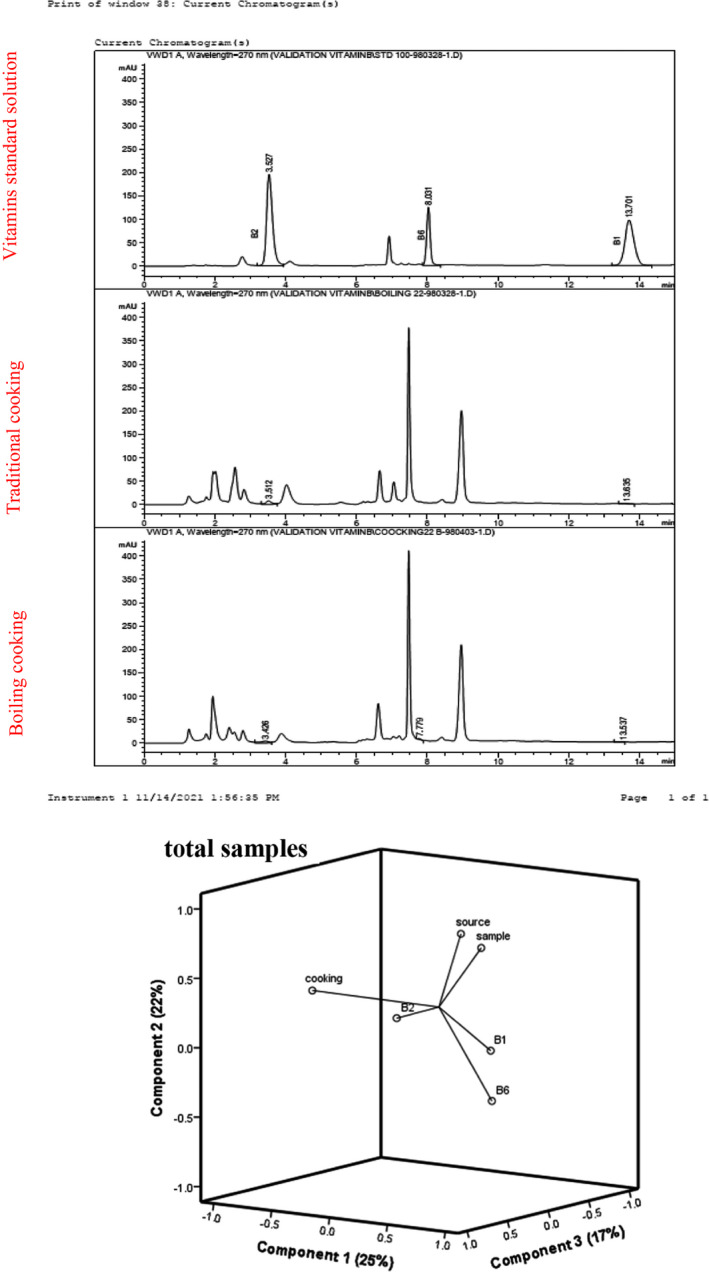
Typical chromatograms of water‐soluble vitamins, standard solution, and rice samples cooked with boiling and traditional cooking methods

Standards and samples were analyzed using a one‐phase reverse (RP‐) HPLC column chromatography that included an autosampler and an 80‐Hz diode array detector (Agilent Technologies, Inc., Santa Rosa, CA). This system was equipped with a 5‐μm Agilent ZORBAX Eclipse Plus C‐18 stationary phase in 4.6 mm × 250 mm formats. The mobile phase of gradient delivery and mobile phase channel A consisted of 5 mmol/L sodium hexane sulfonic acid, 20 mmol/L phosphoric acids, and 16 mmol/L triethylamine (pH 3.0); and mobile phase channel B was mobile phase A: acetic acid, 75:25 (v/v) (pH = 3.54) with a flow rate of 1 ml/min; UV absorption was recorded at 254 nm at 25°C since the vitamin is visible at this wavelength. The standard vitamins B1, B2, and B6 appear at 13.7, 3.52, and 8.03 min retention times, respectively (Figure 1).

### 2.6 The validation of method

The method of validation includes characteristics such as specificity, accuracy, detection limit (LOD), quantitative limit (LOQ), and linearity was determined. For specificity, the analytical solution was made ready by mixing 50 μl of each stock solution in 5 ml of the mobile phase. Two concentrations of analytical solution were made ready by mixing 50 and 5 μl of each stock solution in 10 ml of mobile phase A, respectively (1 and 0.5 μg/ml of each vitamin B compound), for the intra‐ (five times in a day) and interday (3 days), and the precision was assessed by calculating the relative standard deviation (RSD). The LOD was considered using the formula LOD = (3.3 × σ)/S. The intercept of the standard calibration curve was used to calculate the limit of detection (LOD) around the detection limit. Calibration curves were determined by external standardization (0.5, 1, 2.5, 5, 7.5, and 10 μg/ml) of each vitamin B compound.

### 2.7 Recovery assay

Recovery study was carried out based on external addition procedures. Recovery samples were made ready by adding three different levels of each analyte to the matrix. For thiamine and riboflavin, three concentrations of 2, 4, and 8 µg/ml were studied for the recovery assay. In the case of pyridoxine, the selected concentrations were 1, 2, and 4 µg/ml. Recoveries were determined based on the standard method of collection at three different levels. The standard solution for experiments of recovery was prepared by diluting 1 ml of B1, B2, and B6 stock solutions to 10 ml of deionized water. The standard solutions for rice sample recovery tests were prepared by mixing 0.5, 1.0, or 1.5 ml of each solution in 25 ml of deionized water and centrifuged at 1008 g for 5 min. The supernatant was filtered through a micropore filter (0.45 μm) and 20 μl was injected to HPLC. The recoveries were calculated from Equation 1, where A is the peak area of sample spiked with standard, B is the peak area of sample alone (not spiked), and C is the peak area of standard alone (same content used to spike the sample).

(1) Recovery=100%(A‐B)/C

### 2.8 Estimated daily intake (EDI)

The dietary exposures to B1, B2, and B6 vitamins were calculated using the Monte Carlo approach. The EDI (mg/kg day) is applied to quantify the oral exposure dosage for vitamins and estimated by the following Equation (Jahanbakhsh et al., 2019; Rezaei et al., 2019):

(2) $$\text{EDI}=\frac{C\times \text{ED}\times \text{EF}\times \text{IR}}{\text{BW}\times \text{AT}}$$

where EDI is the estimated daily intake (mg/kg day), C is vitamin concentration (mg/kg), IR is ingestion rate (110 g/day), ED is exposure duration (70 years), EF is exposure frequency (365 days/year), BW is reference body mass (the mean weight of children and adults is between 15 and 70 kg, respectively), and AT is the mean time (for both children and adults, it is 25,550 days, respectively) (Jahanbakhsh et al., 2019; Madani‐Tonekaboni et al., 2019; Moradi‐Khatoonabadi et al., 2015; Nikooyeh et al., 2016; Saha & Zaman, 2013).

### 2.9 Data analysis

Statistical analysis was conducted using analysis of variance (ANOVA) and the chi‐square test with SPSS software Ver. 17.0 (SPSS Inc.). The considered significant level was *p* < .05. The association among the type and amount of three vitamins and cooking methods was determined by the multivariate method (Arabameri et al., 2019; Heydarieh et al., 2020). The principal component analysis (PCA) was performed by the SPSS software (Version 18.0). Heatmap analysis was utilized to investigate the correlation among samples (https://biit.cs.ut.ee/clustvis/).

## 3 RESULTS AND DISCUSSION

### 3.1 Residual vitamin content after cooking

As mentioned earlier, B vitamins play a vital role in the metabolism and function of organs. As a result, evaluating the amount of vitamins in consumed foods can have a significant effect on estimating the intake of vitamin balance. Likewise, in this study, we had quantified the remaining B vitamin (B1, B2, and B6) content by the HPLC method in cooked rice samples. The results showed that the remaining content of vitamins B1, B2, and B6 in the traditional samples ranged between 4.09 and 29.55, 4.87 and 16.19, and 1.52 and 12.18 μg/g, respectively (Table 1). However, in the boiling samples, these vitamins had a concentration between 2.98 and 15.89, 1.15 and 22.19, and 0.96 and 4.44 μg/g, respectively. Detection limit (LOD) and quantification limit (LOQ) values were also determined in this study (Table 2). The LOD values for B1, B2, and B6 vitamins were 0.159, 0.090, and 0.041 μg/ml, respectively. Besides, LOQ values were calculated for B1 (0.482 μg/ml), B2 (0.272 μg/ml), and B6 (0.126 μg/ml) vitamins. Considering the sample preparation and dilution procedures, LOQ and LOD of the method presented as μg/g in rice matrix. For B1, B2, and B6, LOQ values were calculated as 9.78, 5.44, and 2.52 μg/g, respectively. LOD values were 3.18, 1.80, and 0.82 μg/g, respectively.

**TABLE 1:** Concentration of B vitamins in a variety of rice samples studied and prepared by different methods

Samples	Sample (*n*)	Traditional (μg/g)			Boiling (μg/g)		
		B1	B2	B6	B1	B2	B6
Ashrafi Gorgan of Iran	3	6.11 ± 0.80	6.8 ± 0.04	3.6 ± 0.30	7.22 ± 0.03	8.91 ± 1.03	3.14 ± 0.4
Fajr Gorgan of Iran	3	6.36 ± 0.90	5.72 ± 0.20	2.64 ± 0.2	11.21 ± 1.00*	8.65 ± 1.1	1.75 ± 0.03
Dom syah fereydunkenar of Iran	3	9.31 ± 1.00	6.27 ± 0.07	3.01 ± 0.05	4.77 ± 1.00	5.69 ± 1.00	1.47 ± 0.06
India Avazeh	3	15.8 ± 1.03*	15.11 ± 1.02*	2.76 ± 0.06	4.83 ± 0.02	14.14 ± 090*	2.36 ± 0.01
Pakistan Shanbeh	3	4.85 ± 0.04	6.70 ± 0.20	2.85 ± 0.01	8.44 ± 1.00	4.27 ± 0.04	1.49 ± 0.01
Dom syah Javaheri of Iran	3	4.58 ± 0.05	5.36 ± 0.04	1.91 ± 0.04	3.56 ± 0.04	6.20 ± 0.04	1.07 ± 0.02
Dom syah dody of Iran	3	4.09 ± 1.00	5.36 ± 0.11	2.14 ± 0.01	15.89 ± 1.10*	9.07 ± 1.00	1.58 ± 0.01
Pakistan Atrak	3	16.59 ± 1.44*	14.13 ± 1.01*	2.59 ± 0.00	7.94 ± 1.40	9.80 ± 1.00	3.45 ± 0.02
Sadry ostokhani of Iran	3	6.02 ± 0.20	11.33 ± 1.22*	3.10 ± 0.51	9.25 ± 0.31	13.76 ± 1.01*	2.70 ± 0.01
Pakistan Mavadat	3	18.67 ± 1.20*	6.21 ± 0.01	12.18 ± 1.00*	2.98 ± 0.2	1.15 ± 0.02	4.44 ± 0.02*
Khanbol Of Iran	3	5.8 ± 0.10	12.23 ± 1.4*	1.52 ± 0.04	2.89 ± 0.01	14.13 ± 0.51*	2.98 ± 0.01
Bab khaneh of Iran	3	29.55 ± 1.50*	7.02 ± 0.80	1.75 ± 0.08	6.39 ± 0.41	1.20 ± 0.01	0.96 ± 0.01
India Mohsen	3	10.07 ± 1.00*	13.27 ± 1.40*	4.66 ± 0.02*	12.48 ± 1.00*	12.03 ± 1.00*	1.24 ± 0.03
Pakistan Azmayesh	3	12.41 ± 1.02*	5.54 ± 0.50	2.32 ± 0.01	6.93 ± 0.01	22.19 ± 2.01*	4.16 ± 0.01*
Pakistan zyton eskandari	3	15.09 ± 1.20*	16.19 ± 1.21*	6.51 ± 0.07*	10.76 ± 1.00*	8.30 ± 1.00	1.68 ± 0.05
Tarem of Iran	3	4.11 ± 0.80	4.87 ± 0.04	3.71 ± 0.02	4.58 ± 0.01	5.46 ± 0.01	1.41 ± 0.01
Hashemi of Iran	3	12.75 ± 1.40*	8.92 ± 1.03	2.33 ± 0.03	4.64 ± 0.02	13.57 ± 1.00*	2.27 ± 0.01
India Golbano	3	5.80 ± 0.10	6.46 ± 0.20	3.42 ± 0.01	6.86 ± 0.01	8.46 ± 0.01	2.98 ± 0.04
India Mojdeh	3	6.04 ± 1.00	5.43 ± 0.84	2.51 ± 0.01	10.65 ± 1.51*	8.22 ± 0.04	1.66 ± 0.04
Anbarbo Dezfol	3	14.73 ± 1.30*	7.83 ± 0.04	2.48 ± 0.02	4.53 ± 0.04	8.62 ± 0.08	3.60 ± 0.01
India Yeganeh	3	5.80 ± 0.04	6.46 ± 0.01	3.42 ± 0.02	4.59 ± 0.04	8.46 ± 0.07	2.98 ± 0.04
Pakistan Momtaz	3	6.04 ± 0.40	5.43 ± 0.24	2.51 ± 0.01	8.02 ± 1.01	8.22 ± 1.01	1.66 ± 0.04

*Denotes significant differences at the 5% level using analysis of variance (ANOVA) based on three repeated determinations.

**TABLE 2:** Method validation parameters

Vitamins	LOD (µg/ml)	LOQ (µg/ml)	*R* ^2^	Concentration (µg/ml)	RSD
B1	0.159	0.482	0.999	0.25	1.303
				0.5	1.430
				1	1.423
				2.5	2.915
				5	3.227
				7.6	1.249
B2	0.090	0.272	0.999	0.25	8.894
				0.5	6.221
				1	9.900
				2.5	2.369
				5	3.619
				7.6	5.242
B6	0.041	0.126	0.999	0.25	0.353
				0.5	1.371
				1	1.886
				2.5	3.505
				5	4.111
				7.6	7.401

Abbreviations: LOD, Limit of detection; LOQ, Limit of quantification; *R* ^2^, *R* Square.

Nevertheless, the use of a traditional cooking method (called “kateh”) is suggested for cooking rice because most of the minerals and vitamins are preserved in this method; the importance of the traditional method has already been reported by several studies (Liu et al., 2019; Mihucz et al., 2010; Shariatifar, Rezaei, Sani, et al., 2020). Typically, soaking and washing are used as conventional precooking processes of rice grains. A considerable decrease in the amount of vitamins and essential elements is usually ascribed to the elimination of the rice husk and bran during the soaking and washing processes, although rinsing of rice can improve cooking quality (Liu et al., 2019).

Shariatifar, Rezaei, Sani, et al. (2020) studied the effect of various cooking methods of rice on the residual mineral content in rice samples and found that the amount of minerals in rice cooked by the rinsing method was less and the rinsing method removes large amounts of minerals. Liu et al. (2019) proved that rice cooking by using the washing method significantly reduced vitamin and mineral contents. Mihucz et al. (2010) also indicated that trace elements, including Cu, Mn, and Zn, are eliminated significantly by the removal of extra water used for soaking and cooking rice samples. Likewise, Jafar et al. (2008) showed that soaking and washing rice samples in warm water before cooking reduce the amount of minerals and nutrients in all studied rice samples. Accordingly, in most of the tested rice, the samples cooked by the traditional method had almost higher vitamin content than the boiling method. Because food processing (like cooking) has the potential to change the thermal stability of vitamins or other nutraceuticals in food products, some of the vitamins may be wasted during rinsing due to their water solubility.

Nevertheless, the thermal stability of vitamins can be effective in reducing their loss. As reported previously, thiamine was the most heat‐sensitive vitamin, while vitamins B2 and B6 were more thermally stable (Bui et al., 2013; Fuliaş et al., 2014; Liberato & Pinheiro‐Sant'Ana, 2006). However, riboflavin possessed the highest thermal stability (Kim et al., 2013; Kwok et al., 1998). Among the vitamins studied, the lowest concentration was related to vitamin B6, while vitamins B1 and B2 had nearly the same concentration range. Consequently, this finding indicates that rice can be considered a very good source of B‐group vitamins. In this regard, many studies have investigated the thermal stability of B‐group vitamins. Fuliaş et al. (2014) studied the thermal stability of B1, B2, and B6 vitamins under dynamic air atmosphere and nonisothermal conditions, and found riboflavin to be the most stable vitamin. They reported that the high thermal stability of vitamin B2 can be attributed to the presence of the N‐heterocyclic aromatic benzo[g]pteridine moiety and carbohydrate moiety in the molecular structure, which is known to be a stabilizing structure. Moreover, the degree of aromaticity of vitamin B2 is significantly higher than that of B1 and B6. Similar to the single‐bonded type, double‐bonded nitrogen and/or oxygen can also be part of a conjugated structure owing to the lone pair orbital, which can become part of an expanded conjugated aromatic system.

Liu et al. (2019) indicated that rice cooking significantly reduced B1 and B2 contents, which are consistent with the findings of our study. In another study, Kim et al. (2013) measured the water‐soluble vitamins content at the various thermal processing phases in garlic samples. They reported that the amount of water‐soluble vitamins decreased at various temperature stages (Kim et al., 2013). Papastoyiannidis et al. (2006) investigated the vitamins’ thermal stability in fermented milk fortified with B‐group vitamins (B1, B2, B6, and folic acid). The vitamin concentration of fortified milk was reduced after heating at 77–78°C for 50 min.

### 3.2 Structural relationship of parameters

For a better understanding of potential quality deterioration among the prepared samples (by different cooking methods), the principal component analysis (PCA) multivariate analysis was performed using the SPSS software. Multivariate method was conducted to evaluate the correlation between the type and content of vitamins and cooking methods.

**FIGURE 2:**
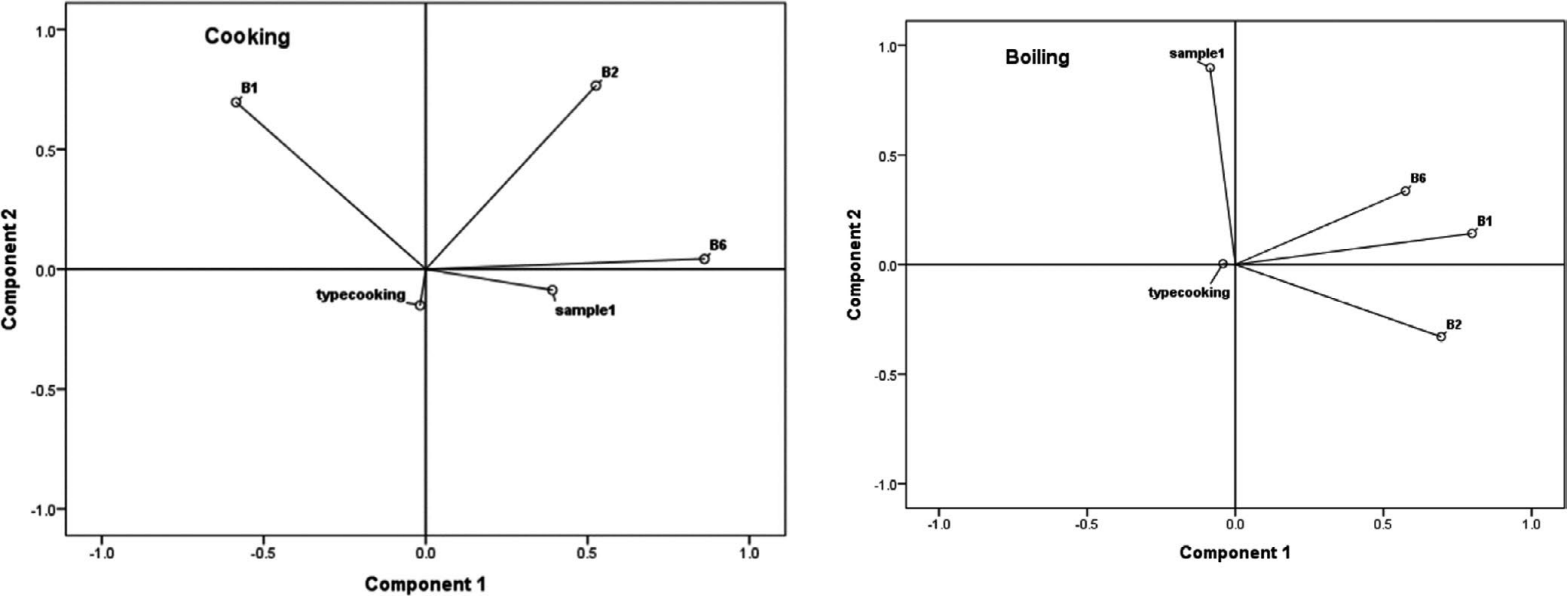
Principal component analysis plot of thiamin, riboflavin, and B6 in rice samples by different cooking methods

**FIGURE 3:**
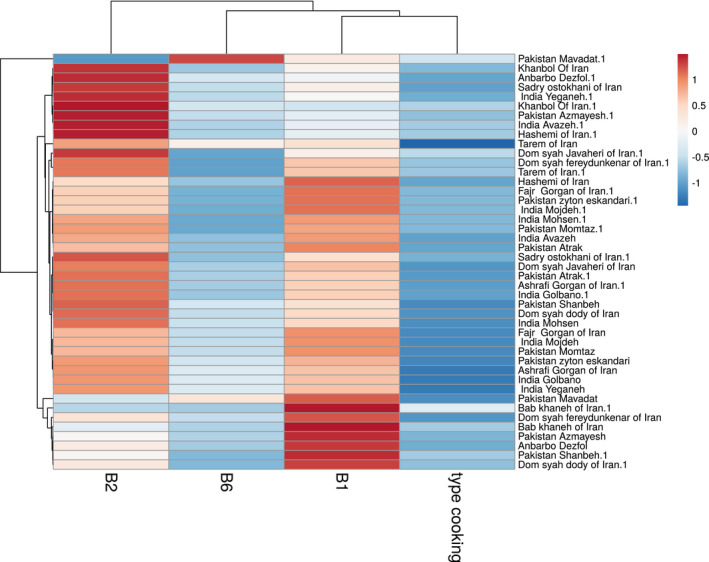
Heatmap of three vitamins and types of cooking methods in rice samples

Clust‐Vis was applied to visualize clustering of similarity and variability data (Figures 2 and 3). The three vitamin and cooking method heatmap clustered 46 rice samples into two major clusters and two subclusters (Figure 3). The first cluster presents only riboflavin, and the second cluster includes two subgroups with B2 and B6, and cooking methods. This showed that the B1 and B6 vitamins in rice samples were more sensitive than vitamin B2 in the cooking method used. In this regard, many studies used multivariate statistical methods to analyze data of nutritional quality and thermal stability of food products (Guo et al., 2020; Heydarieh et al., 2020; Shariatifar et al., 2020). Multivariate statistical methods revealed a detailed relationship among reduced B‐group vitamins and rice cooking in different groups.

Quantitative results obtained for the two cooking methods were used in PCA to investigate the potential quality deterioration among the prepared samples (by different cooking methods). The compounds included sample type, source location, different cooking methods, B1, B2, and B6 vitamins.

The correlation among various cooking methods can be observed from Figure 2; the plot subset shows a positive correlation between various cooking methods. Nearest‐neighbor analysis of the characteristics revealed a considerable association between dependent variables.

As revealed in Figure 2, the first three principal components accounted for 66.38% of the data variance in all samples, and their contribution rates were 25.84%, 22.67%, and 17.86%, respectively. B1 and B6 vitamins were the closest accessions. The B1, B2, and B6 vitamins, sample types, and source location had a high positive correlation with PC1, while they had a negative correlation with cooking methods. The results showed that the sample type and source location had a positive correlation with PC2, while they had a negative correlation with vitamin B6.

In the cooking samples, the first, second, and third components accounted for34.788%, 28.50%, and 20.3% of the total variability, respectively. The first two principal components explained 63.2% of the data variance.

Thiamin and source location showed a high positive correlation with PC1, whereas a negative correlation was seen with B6 vitamin. The results showed that the sample type, source location, different cooking methods, B1, B2, and B6 vitamins indicated a positive correlation with PC2, although a negative correlation was observed with B1 vitamin. The first and second principal components described 29.19% and 26.28% of the total variability in the boiling samples. The PCA patterns visualized relationships between cooking methods and B1, B2, and B6 vitamin levels, which indicates that they are important factors for studying the effects of household cooking processes on B‐group vitamins in rice samples.

### 3.3 Dietary exposure estimates

**FIGURE 4:**
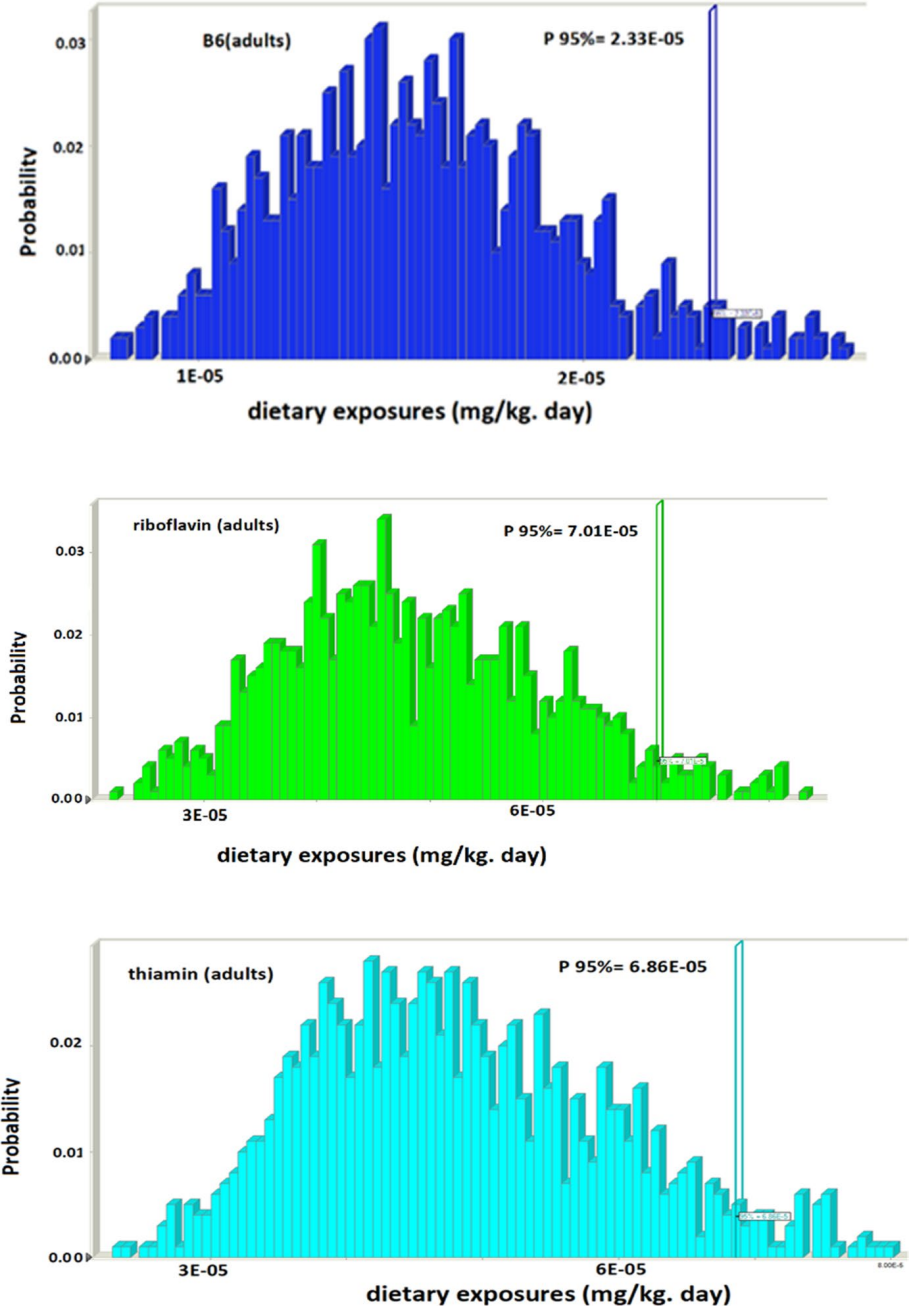
Simulation results for dietary exposures (mg/kg day) of thiamin, riboflavin, and pyridoxine in rice samples

The mean, minimum, and maximum dietary exposures to three vitamins from different rice varieties cooked are presented in Table 3. Simulation results for dietary exposures (mg/kg day) of thiamin, riboflavin, and pyridoxine in rice samples are also demonstrated in Figure 4. The mean exposure levels of B1, B2, and B6 vitamin for adults were 2.05E‐05, 1.94E‐05, and 6.59E‐06 (mg/kg ·BW ·day), respectively; and for children, these were 6.86E‐05, 7.01E‐05, and 2.32E‐05, respectively, at the extreme percentile (P95), which are far below ADIs (1, 1.1, and 1.1 mg/kg ·BW ·day, respectively). Monitoring the levels of essential trace elements in food products is important; the knowledge of estimated daily intake of B1, B2, and B6 vitamins via rice is equally crucial and has been a topic of many experimental studies. In this regard, Sumczynski et al. (2018) studied the vitamin B compound contents and their dietary intake of rice flakes in commercial wild rice samples. The findings of their study revealed that wild rice flakes are a significant source to the RDA of niacin (28%) and thiamine (31%) (Sumczynski et al., 2018).

**TABLE 3:** The mean, minimum, and maximum dietary exposures (mg/kg d) of three vitamins from different rice varieties cooked

Vitamins	Percentiles	Ears (mg/day)	Adults (mg/kg day)				Children (mg/kg day)			
			5%	50%	75%	95%	5%	50%	75%	95%
B1	Mean	1	9.17E‐6	1.34E‐5	1.58E‐5	2.05E‐5	3.22E‐5	4.68E‐5	5.53E‐5	6.86E‐5
	Minimum		3.06E‐6	4.53E‐6	5.39E‐6	6.84E‐6	1.05E‐5	1.59E‐5	1.88E‐5	2.36E‐5
	Maximum		3.14E‐5	4.59E‐5	5.38E‐5	6.90E‐5	1.09E‐4	1.63E‐4	1.91E‐4	2.43E‐4
B2	Mean	1.1	8.89E‐6	1.34E‐5	1.56E‐5	1.94E‐5	3.13E‐5	4.72E‐5	5.67E‐5	7.01E‐5
	Minimum		1.17E‐6	1.83E‐6	2.13E‐6	2.65E‐6	4.19E‐6	6.31E‐6	7.44E‐6	9.34E‐6
	Maximum		2.31E‐5	3.42E‐5	4.05E‐5	5.12E‐5	8.20E‐5	1.21E‐4	1.43E‐4	1.85E‐4
B6	Mean	1.1	2.94E‐6	4.44E‐6	5.24E‐6	6.59E‐6	1.05E‐5	1.55E‐5	1.83E‐5	2.32E‐5
	Minimum		1.01E‐6	1.50E‐6	1.77E‐6	2.18E‐6	3.56E‐6	5.26E‐6	6.22E‐6	7.54E‐6
	Maximum		1.30E‐5	1.89E‐5	2.22E‐5	2.76E‐5	4.45E‐5	6.62E‐5	7.89E‐5	1.01E‐4

## 4 CONCLUSION

To summarize, as proved in this study, processes such as precooking and cooking methods can have a remarkable effect on the amount of minerals, vitamins, and other nutrients in rice. In this study, the remaining content of vitamins (B1, B2, and B6) in rice samples cooked by rinsing and the conventional method was detected by the HPLC method. The amount of B1, B2, and B6 vitamins ranged from 4.09 to 29.55, 4.87 to 16.19, and 1.52 to 12.18 μg/g for the traditional samples, respectively. In the boiling (soaking) cooking method, these vitamins had a concentration between 2.89 and 15.89, 1.15 and 22.19, and 0.96 and 4.44 μg/g, respectively. However, LOD values for B1, B2, and B6 vitamins were obtained at 0.159, 0.090, and 0.041 μg/ml, respectively. From these results, it can be inferred that the conventional cooking method can preserve more vitamins and minerals in rice samples because a lot of these nutrients are wasted in the rinsing method when washing and soaking.

The correlation investigation focuses on the numerical association between studied vitamins under all conditions. Comparing relationships among thiamin, riboflavin, and vitamin B6 from different rice varieties cooked showed that these vitamin contents can change. Additionally, the results of heat map visualization showed that the association among the three vitamins was clearly separated according to the cooking process. Higher dietary intakes of soluble vitamins were associated with thiamin (2.05E‐05, mg/kg BW day).

## CONFLICT OF INTEREST

The authors declare that they have no conflict of interest.

## ETHICAL APPROVAL

This article does not contain any studies with participants or animals requiring ethical approval.

## Data Availability

The data that support the findings of this study are available from the corresponding author upon reasonable request.

## References

[fsn32690-bib-0001] Arabameri, M. , Nazari, R. R. , Abdolshahi, A. , Abdollahzadeh, M. , Mirzamohammadi, S. , Shariatifar, N. , Barba, F. J. , & Mousavi Khaneghah, A. (2019). Oxidative stability of virgin olive oil: Evaluation and prediction with an adaptive neuro‐fuzzy inference system (ANFIS). Journal of the Science of Food and Agriculture, 99(12), 5358–5367. 31056745 10.1002/jsfa.9777

[fsn32690-bib-0002] Bresciani, A. , Pagani, M. A. , & Marti, A. (2021). Rice: A versatile food at the heart of the mediterranean diet. In F. Boukid (Ed.), Cereal‐Based Foodstuffs: The Backbone of Mediterranean Cuisine (pp. 193–229). Springer.

[fsn32690-bib-0003] Bui, L. T. , Small, D. M. , & Coad, R. (2013). The stability of water‐soluble vitamins and issues in the fortification of foods. In Handbook of Food Fortification and Health (pp. 199–211). Humana Press.

[fsn32690-bib-0004] Cheigh, H. , Ryu, C. , Jo, J. , & Kwon, T. (1977). A type of post‐harvest loss: Nutritional losses during washing and cooking of rice. Korean Journal of Food Science and Technology, 9(3), 229–233.

[fsn32690-bib-0005] de Souza Batista, C. , dos Santos, J. P. , Dittgen, C. L. , Colussi, R. , Bassinello, P. Z. , Elias, M. C. , & Vanier, N. L. (2019). Impact of cooking temperature on the quality of quick cooking brown rice. Food Chemistry, 286, 98–105. 30827672 10.1016/j.foodchem.2019.01.187

[fsn32690-bib-0006] Descombes, E. , Hanck, A. B. , & Fellay, G. (1993). Water soluble vitamins in chronic hemodialysis patients and need for supplementation. Kidney International, 43(6), 1319–1328. 8315945 10.1038/ki.1993.185

[fsn32690-bib-0007] Fuliaş, A. , Vlase, G. , Vlase, T. , Oneţiu, D. , Doca, N. , & Ledeţi, I. (2014). Thermal degradation of B‐group vitamins: B 1, B 2 and B 6. Journal of Thermal Analysis and Calorimetry, 118(2), 1033–1038.

[fsn32690-bib-0008] Galán, M. G. , & Drago, S. R. (2014). Food matrix and cooking process affect mineral bioaccessibility of enteral nutrition formulas. Journal of the Science of Food and Agriculture, 94(3), 515–521. 23794294 10.1002/jsfa.6280

[fsn32690-bib-0009] Guo, C. , Xie, Y.‐J. , Zhu, M.‐T. , Xiong, Q. , Chen, Y. , Yu, Q. , & Xie, J.‐H. (2020). Influence of different cooking methods on the nutritional and potentially harmful components of peanuts. Food Chemistry, 316, 126269. 10.1016/j.foodchem.2020.126269 32044701

[fsn32690-bib-0010] Heydarieh, A. , Arabameri, M. , Ebrahimi, A. , Ashabi, A. , Monjazeb Marvdashti, L. , Shokrollahi Yancheshmeh, B. , & Abdolshahi, A. (2020). Determination of magnesium, calcium and sulphate ion impurities in commercial edible salt. Journal of Chemical Health Risks, 10(2), 93–102.

[fsn32690-bib-0011] Jafar, M. , Basem, F. D. , Maha, M. , & Khalil, I. E. (2008). Variation in physio‐chemical characteristics, mineral concentrations and cookability of rice marketed in Jordan. Pakistan Journal of Nutrition, 7(1), 141–145.

[fsn32690-bib-0012] Jahanbakhsh, M. , Afshar, A. , Momeni Feeli, S. , Pabast, M. , Ebrahimi, T. , Mirzaei, M. , Akbari‐Adergani, B. , & Farid, M. & Arabameri, M. (2019). Probabilistic health risk assessment (Monte Carlo simulation method) and prevalence of aflatoxin B1 in wheat flours of Iran. International Journal of Environmental Analytical Chemistry, 101(8), 1074–1085. 10.1080/03067319.2019.1676421

[fsn32690-bib-0013] Kim, J.‐S. , Kang, O.‐J. , & Gweon, O.‐C. (2013). Changes in the content of fat‐and water‐soluble vitamins in black garlic at the different thermal processing steps. Food Science and Biotechnology, 22(1), 283–287.

[fsn32690-bib-0014] Kwok, K. C. , Shiu, Y. W. , Yeung, C. H. , & Niranjan, K. (1998). Effect of thermal processing on available lysine, thiamine and riboflavin content in soymilk. Journal of the Science of Food and Agriculture, 77(4), 473–478.

[fsn32690-bib-0015] Liberato, S. C. , & Pinheiro‐Sant'Ana, H. M. (2006). Fortification of industrialized foods with vitamins. Revista de Nutrição, 19(2), 215–231.

[fsn32690-bib-0016] Liu, K.‐L. , Zheng, J.‐B. , & Chen, F.‐S. (2017). Relationships between degree of milling and loss of Vitamin B, minerals, and change in amino acid composition of brown rice. LWT‐Food Science and Technology, 82, 429–436.

[fsn32690-bib-0017] Liu, K. , Zheng, J. , Wang, X. , & Chen, F. (2019). Effects of household cooking processes on mineral, vitamin B, and phytic acid contents and mineral bioaccessibility in rice. Food Chemistry, 280, 59–64. 30642507 10.1016/j.foodchem.2018.12.053

[fsn32690-bib-0018] Madani‐Tonekaboni, M. , Sadat aghayan, N. , Rafiei Nazari, R. , Mirzamohammadi, S. , Abdolshahi, A. , Abbasi‐bastami, N. , & Arabameri, M. (2019). Monitoring and risk assessment of lead and cadmium in milks from east of Iran using Monte Carlo simulation method. Nutrition and Food Sciences Research, 6(2), 29–36. 10.29252/nfsr.6.2.29

[fsn32690-bib-0019] Mahan, L. K. , & Raymond, J. L. (2016). Krause's food & the nutrition care process, Mea Edition E‐Book: Elsevier.

[fsn32690-bib-0020] Mihucz, V. G. , Silversmit, G. , Szalóki, I. , Samber, B. D. , Schoonjans, T. , Tatár, E. , Vincze, L. , Virág, I. , Yao, J. , & Záray, G. (2010). Removal of some elements from washed and cooked rice studied by inductively coupled plasma mass spectrometry and synchrotron based confocal micro‐X‐ray fluorescence. Food Chemistry, 121(1), 290–297.

[fsn32690-bib-0021] Moradi‐Khatoonabadi, Z. , Amirpour, M. , & AkbariAzam, M. (2015). Synthetic food colours in saffron solutions, saffron rice and saffron chicken from restaurants in Tehran, Iran. Food Additives & Contaminants: Part B, 8(1), 12–17. 10.1080/19393210.2014.945195 25116149

[fsn32690-bib-0022] Nikooyeh, B. , Abdollahi, Z. , Salehi, F. , Nourisaeidlou, S. , Hajifaraji, M. , Zahedirad, M. , Shariatzadeh, N. , Kalayi, A. , Babaei balderlou, F. , Gholizadeh salmasi, J. , Entezarmahdi, R. , Ghorbannezhad, Z. , Lotfollahi, N. , Maleki, M.‐R. , & Neyestani, T. R. (2016). Prevalence of obesity and overweight and its associated factors in urban adults from West Azerbaijan, Iran: The National Food and Nutritional Surveillance Program (NFNSP). Nutrition and Food Sciences Research, 3(2), 21–26.

[fsn32690-bib-0023] Papastoyiannidis, G. , Polychroniadou, A. , Michaelidou, A.‐M. , & Alichanidis, E. (2006). Fermented milks fortified with B‐group vitamins: Vitamin stability and effect on resulting products. Food Science and Technology International, 12(6), 521–529.

[fsn32690-bib-0024] Rezaei, M. , Ghasemidehkordi, B. , Peykarestan, B. , Shariatifar, N. , Jafari, M. , Fakhri, Y. , Jabbari, M. & Khaneghah, A. M. (2019). Potentially toxic element concentration in fruits collected from Markazi Province (Iran): A probabilistic health risk assessment. Biomedical and Environmental Sciences, 32(11), 839–853. 31910941 10.3967/bes2019.105

[fsn32690-bib-0025] Saha, N. , & Zaman, M. (2013). Evaluation of possible health risks of heavy metals by consumption of foodstuffs available in the central market of Rajshahi City, Bangladesh. Environmental Monitoring and Assessment, 185(5), 3867–3878. 22933105 10.1007/s10661-012-2835-2

[fsn32690-bib-0026] Shariatifar, N. , Rezaei, M. , Alizadeh Sani, M. , Alimohammadi, M. , & Arabameri, M. (2020). Assessment of rice marketed in iran with emphasis on toxic and essential elements; effect of different cooking methods. Biological Trace Element Research, 198(2), 721–731. 10.1007/s12011-020-02110-1 32189243

[fsn32690-bib-0027] Shariatifar, N. , Rezaei, M. , Sani, M. A. , Alimohammadi, M. , & Arabameri, M. (2020). Assessment of rice marketed in Iran with emphasis on toxic and essential elements; effect of different cooking methods. Biological Trace Element Research, 1–11. 10.1007/s12011-020-02110-1 32189243

[fsn32690-bib-0028] Sumczynski, D. , Koubová, E. , Šenkárová, L. , & Orsavová, J. (2018). Rice flakes produced from commercial wild rice: Chemical compositions, vitamin B compounds, mineral and trace element contents and their dietary intake evaluation. Food Chemistry, 264, 386–392. 29853391 10.1016/j.foodchem.2018.05.061

[fsn32690-bib-0029] Verma, D. K. , & Srivastav, P. P. (2020). Bioactive compounds of rice (Oryza sativa L.): Review on paradigm and its potential benefit in human health. Trends in Food Science & Technology, 97, 355–365.

[fsn32690-bib-0030] Zhu, L. , Wu, G. , Cheng, L. , Zhang, H. , Wang, L. , Qian, H. , & Qi, X. (2019). Effect of soaking and cooking on structure formation of cooked rice through thermal properties, dynamic viscoelasticity, and enzyme activity. Food Chemistry, 289, 616–624. 30955656 10.1016/j.foodchem.2019.03.082

